# Supplementary material for: Treatment of Marmoset Intracerebral Hemorrhage with Humanized Anti-HMGB1 mAb
Source: Cells. 2022 Sep 23;11(19):2970. doi: 10.3390/cells11192970 (PMC9563572; doi:10.3390/cells11192970)
Supplement: Supplementary file 1 [file cells-11-02970-s001.zip › cells-1904408-supplementary.pdf]

Treatment of Marmoset Intracerebral Hemorrhage with Humanized Anti-HMGB1 mAb

Dengli Wang [1], Daiki Ousaka [1], Handong Qiao [1], Ziyi Wang [2,3], Kun Zhao [3], Shangze Gao [4], Keyue Liu [1], Kiyoshi Teshigawara [1], Kenzo Takada [5] and Masahiro Nishibori [6,*]

[1] Department of Pharmacology, Faculty of Medicine, Dentistry, and Pharmaceutical Sciences, Okayama University, Okayama, 7008558, Japan
[2] Research Fellow of Japan Society for the Promotion of Science, Tokyo, 1020083, Japan
[3] Department of Molecular Biology and Biochemistry, Faculty of Medicine, Dentistry and Pharmaceutical Sciences, Okayama University, Okayama, 7008558, Japan
[4] School of Pharmaceutical Sciences, Tsinghua University, Beijing, China
[5] Sapporo Laboratory, EVEC, Inc., Sapporo, 0606642, Japan
[6] Department of Translational Research and Drug Development, Faculty of Medicine, Dentistry and Pharmaceutical Sciences, Okayama University, Okayama, 7008558, Japan
* Correspondence: mbori@md.okayama-u.ac.jp

Supplemental Figures

Figure S1

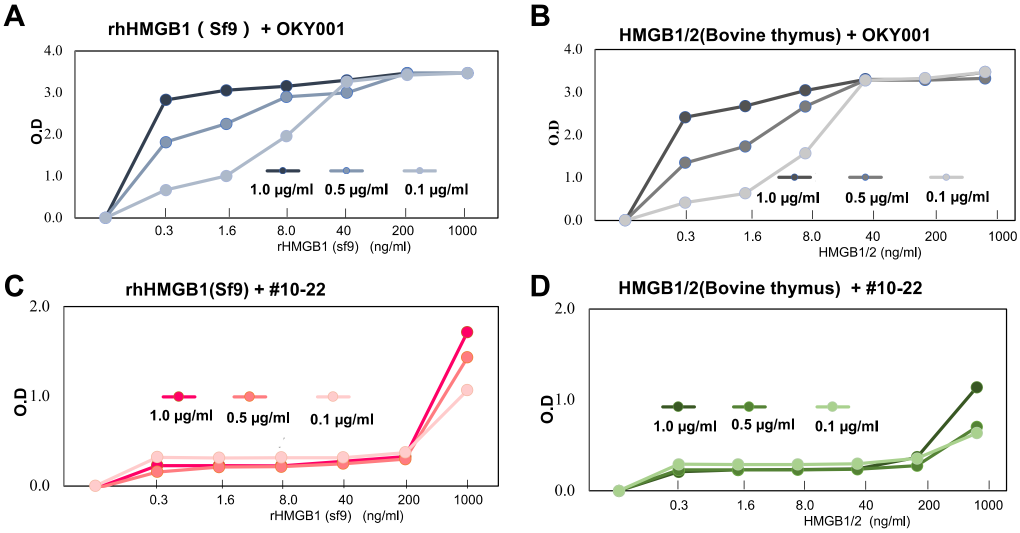

Figure S1**:** The detection efficiency of humanized anti-HMGB1 mAb (OKY-001) compared with the original rat mAb(#10-22) by Elisa assay. **A.** Different amounts of recombinant HMGB1 were used to detect the efficiency of humanized anti-HMGB1 mAb (OKY-001). **B.** Different amounts of native HMGB1 were used to detect the efficiency of humanized anti-HMGB1 mAb (OKY-001). **C.** Different amounts of recombinant HMGB1 were used to detect the efficiency of rat anti-HMGB1 mAb (#10-22).**D.** Different amounts of native HMGB1 were used to detect the efficiency of rat anti-HMGB1 mAb (#10-22).

Figure S2

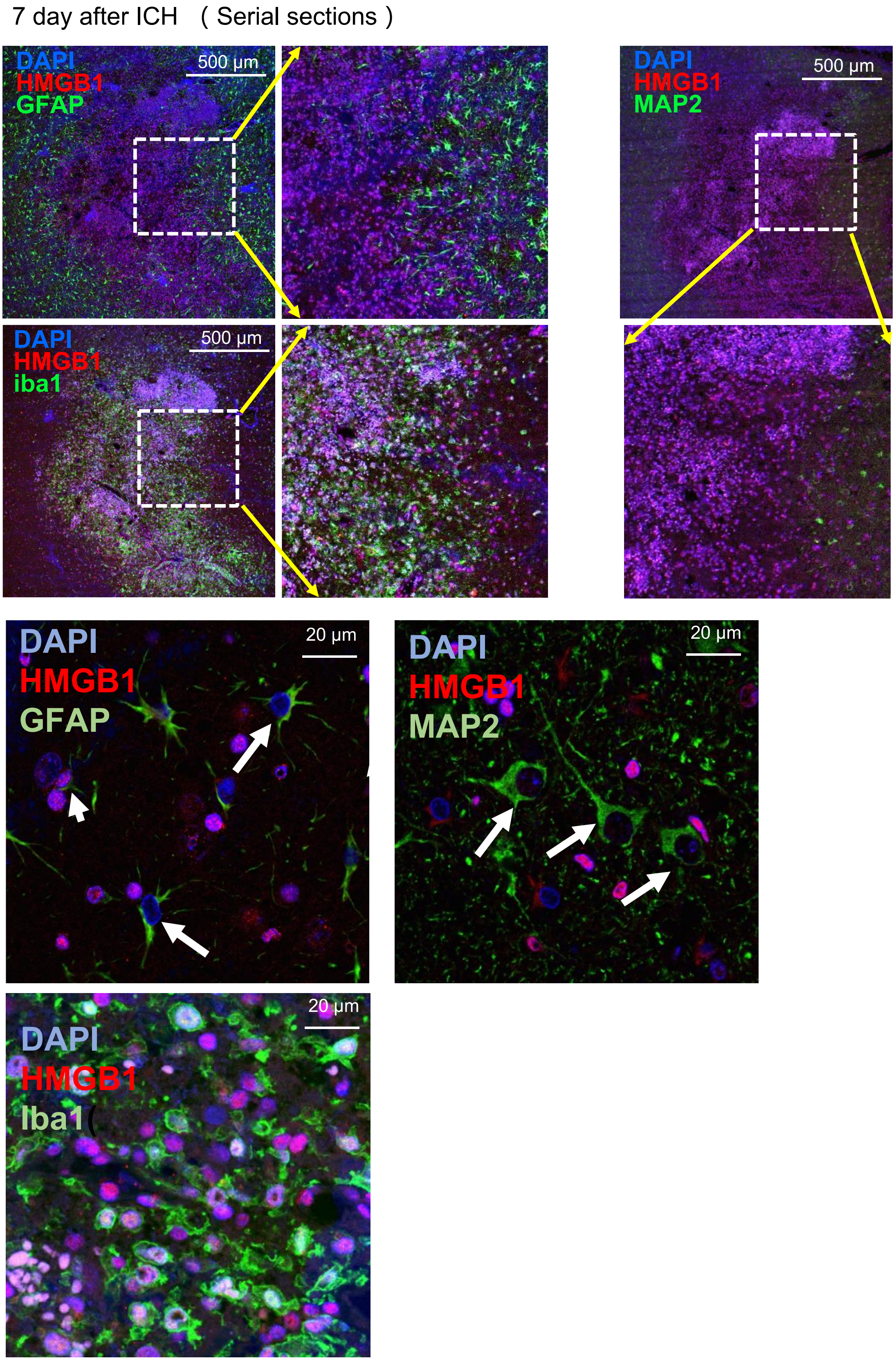

Figure S2: Representative images of the immunostained brain serial sections at 7 d after ICH. The expressions of HMGB1 (red) with GFAP (green), ibal1 (green) and MAP2 (green) are shown in the hematoma zone at 7 d after establishing ICH in a marmoset.

Figure S3

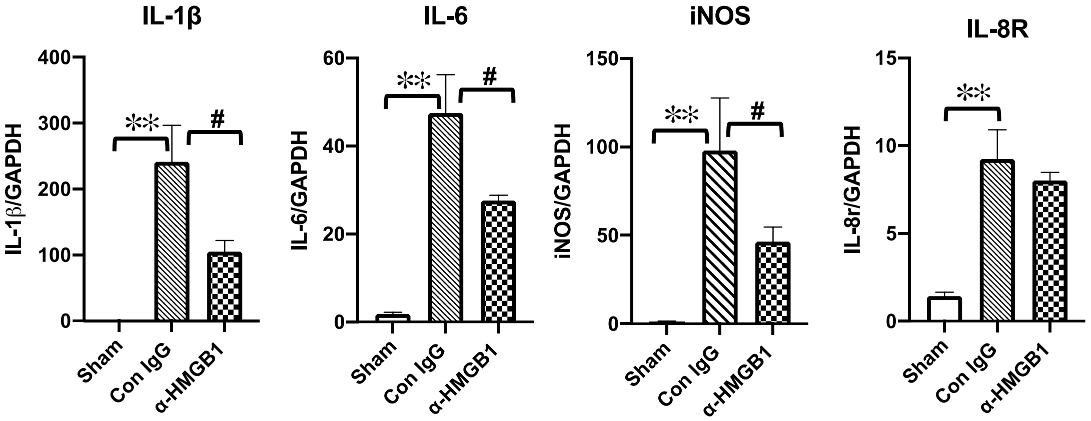

Figure S3: The expressions of interleukin-1β (IL-1β), IL-6, inducible nitric oxide synthase (iNOS), and IL-8R were measured at 24 h after ICH in the rat brain with or without treatment of humanized anti-HMGB1 mAb. The brain samples for qRT-PCR were obtained from peri-hematomal regions (each sample weighed about 25 mg). GAPDH expression was used as an internal control to normalize cDNA levels. Fold changes in expression levels were calculated by the comparative cycle threshold method ($2^{-\Delta\Delta CT}$). ***p* < 0.01 compared with sham values. #*p* < 0.05 compared with Con IgG values.
